# Barriers and facilitating factors for disease self-management: a qualitative analysis of perceptions of patients receiving care for type 2 diabetes and/or hypertension in San José, Costa Rica and Tuxtla Gutiérrez, Mexico

**DOI:** 10.1186/1471-2296-14-131

**Published:** 2013-09-04

**Authors:** Meredith P Fort, Nadia Alvarado-Molina, Liz Peña, Carlos Mendoza Montano, Sandra Murrillo, Homero Martínez

**Affiliations:** 1 Comprehensive Center for the Prevention of Chronic Diseases (CIIPEC), Institute of Nutrition of Central America and Panamá, Calzada Roosevelt 6-25, Zona 11, Guatemala City, Guatemala; 2 School of Nutrition, University of Costa Rica, San José, Costa Rica; 3 School of Nutrition, University of Arts and Sciences in Chiapas, Tuxtla Gutiérrez, Chiapas, Mexico; 4 RAND Corporation, 1776 Main Street, Santa Mónica, California, USA; 5 Hospital Infantil de México “Dr. Federico Gómez”, Mexico City, Mexico

**Keywords:** Cardiovascular health, Trans-theoretical model, Health promotion, Primary care

## Abstract

**Background:**

The burden of cardiovascular disease is growing in the Mesoamerican region. Patients’ disease self-management is an important contributor to control of cardiovascular disease. Few studies have explored factors that facilitate and inhibit disease self-management in patients with type 2 diabetes and hypertension in urban settings in the region. This article presents patients’ perceptions of barriers and facilitating factors to disease self-management, and offers considerations for health care professionals in how to support them.

**Methods:**

In 2011, 12 focus groups were conducted with a total of 70 adults with type 2 diabetes and/or hypertension who attended urban public health centers in San José, Costa Rica and Tuxtla Gutiérrez, Chiapas, Mexico. Focus group discussions were transcribed and coded using a content analysis approach to identify themes. Themes were organized using the trans-theoretical model, and other themes that transcend the individual level were also considered.

**Results:**

Patients were at different stages in their readiness-to-change, and barriers and facilitating factors are presented for each stage. Barriers to disease self-management included: not accepting the disease, lack of information about symptoms, vertical communication between providers and patients, difficulty negotiating work and health care commitments, perception of healthy food as expensive or not filling, difficulty adhering to treatment and weight loss plans, additional health complications, and health care becoming monotonous. Factors facilitating disease self-management included: a family member’s positive experience, sense of urgency, accessible health care services and guidance from providers, inclusive communication, and family and community support.

Financial difficulty, gender roles, differences by disease type, faith, and implications for families and their support were identified as cross-cutting themes that may add an additional layer of complexity to disease management at any stage. These factors also relate to the broader family and societal context in which patients live.

**Conclusions:**

People living with type 2 diabetes and hypertension present different barriers and facilitating factors for disease self-management, in part based on their readiness-to-change and also due to the broader context in which they live. Primary care providers can work with individuals to support self-management taking into consideration these different factors and the unique situation of each patient.

## Background

Chronic diseases currently account for the largest share of morbidity and mortality in most of the developing world. More than 80% of deaths from chronic disease now occur in low and middle-income countries, [1,2] and they represent a substantial burden for individuals, families, and national economies [3]. Caring for these diseases places a burden on the affected individual and family members, for often the disease goes unnoticed for a long period, and by the time it gets diagnosed, there may be complications present that can be incapacitating or life-threatening [4]. At the same time, the primary health care system, often overburdened by other acute and chronic conditions, may not be prepared to properly deal with patients presenting these conditions [5]. In low and middle-income countries, cardiovascular diseases have become the most frequent cause of death and disease burden [6-8]. Arterial hypertension and type 2 diabetes, the conditions emphasized in this article, are leading causes of death, disability, and represent a major cost to health care systems in Central America and Mexico [9,10].

There is strong evidence indicating that proper management and care for these two illnesses should not be based only on pharmaceutical treatment, but also through the adoption of healthy lifestyle by patients [11,12]. Patient perceptions of disease and effective communication between patients and health care providers plays an important role in the promotion of lifestyle change and chronic disease management [13]. Patient denial and non-adherence to hypertension treatment is a prevalent phenomenon reflecting a conscious choice made by the patient, based on his knowledge and perceptions regarding the medical condition and its treatment [14]. Individuals who perceive their blood pressure as uncontrolled may have intentions to make health-enhancing changes but may lack the information to do so [15]. Fatalism is quite common among patients diagnosed with diabetes and is associated with poor medication adherence and self-care and may be an important target for education and skills interventions in diabetes care [16].

A number of authors call on primary care providers to take a more patient-centered approach to those with diabetes and hypertension. Some ways to do this are by reducing wait times, encouraging patient self-management by developing a patient version of care guidelines, and increasing educational efforts [17]. One model focused on patients that has been used in health promotion interventions in primary care settings is the transtheoretical model of change, originally explained by Prochaska and Diclemente in the 1980s. The model proposes that behavior change is experienced as a series of stages and can be useful in offering an understanding of the extent to which patients are able to make change.

There are mixed results about the extent to which stage-based care results in improved health care outcomes for patients with chronic conditions, but authors have identified it as being useful for individualizing care plans and improving patient-provider communication [18]. In a systematic review assessing the effectiveness of transtheoretical model-based dietary and physical activity interventions on weight loss in overweight and obese adults, minimal weight loss was observed, but there was no evidence of it being sustained; the authors conclude that the framework in combination with other strategies may result in improved outcomes [19].

The health care infrastructure and context in which these patients receive their care, and the family and broader social environment in which they live can also be important factors influencing patients’ perceptions. As such, we offer a brief background on the two settings in which this study was conducted.

During the last few decades, Mexico has undergone changes in its population structure and epidemiologic trends resulting in a health profile characterized by the predominance of non-communicable diseases such as hypertension and type 2 diabetes [20]. Based on an analysis of the Mexican National Health and Nutrition Survey, 43% of Mexican adults have hypertension [21] and the prevalence of type 2 diabetes in Mexican adults is 14.4% [22].

While the Mexican health care system includes different institutions, the Secretary of Health has the mandate to provide health care to the whole population, specifically for those workers and direct dependents who are not regularly employed (as the formal working population is covered by the Social Security system). Our study takes place in Secretary of Health primary care centers in Tuxtla Gutiérrez, the capital city of the state of Chiapas, one of the least developed states in terms of health resources and infrastructure, with a population of a half a million people. These centers are typically staffed with two family physicians, a dentist, a dietician, nurses and social workers. There is also a community health worker who is in charge of health promotion programs. Medications are provided to patients with diabetes and hypertension free-of-charge at health centers with a maximum 30-day supply.

Costa Rica has long been recognized in the Mesoamerican region for its comprehensive health care system, and health outcomes that are similar to those achieved in many high-income countries [23]. Currently non-communicable diseases represent the major disease burden in the country. Over 1.5 million people live in the wider San José area, the capital city. A survey conducted in 2004 in San José estimated the following disease prevalence: type 2 diabetes (7.9%), hypertension (25.6%), and pre-hypertension (25.5%) [24].

For half a century the Costa Rican Social Security Institute (CCSS) has been in charge of financing and providing services to the population. Over 90% of the population receives care from the CCSS. The need to improve service and lower costs by strengthening the primary health care system was recognized in 1996 and a new system of care was put in place called EBAIS (Basic Health Attention Teams) responsible for a community’s physical and social needs [25]. Each EBAIS has at a minimum: one general practice physician, a nurse’s assistant, a primary health care technical assistant, and a medical registry assistant. The EBAIS offers a full range of primary care, health promotion, and preventive services that are free at the point of service delivery [26].

Medications are free-of-charge for patients with diabetes and hypertension and laboratory tests and primary health care clinic visits are scheduled according to disease type and progression; people with hypertension are scheduled once every 4 months and patients with type 2 diabetes are scheduled every 3 months, and the highest risk patients have consultations more frequently.

The main purpose of this article is to present how patients with type 2 diabetes and/or hypertension from Tuxtla Gutiérrez, Chiapas, Mexico and San José, Costa Rica perceive their role in managing their disease, and summarize primary facilitating factors and barriers. The TransTheoretical Model is used as a way of categorizing patients’ barriers and facilitators to care. This paper contributes to the existing literature on patient perceptions of chronic care self-management in two populations in the Mesoamerican region, a setting with limited published studies on this topic. This study may be of particular interest to health care providers by offering new perspectives on how to support patients with diabetes and hypertension, based on patients’ views.

## Methods

From July to November in 2011, focus group discussions (FGDs) with patients with hypertension and diabetes were carried out as part of the formative research phase for a project titled “Primary health care and community support model to reduce the risk of cardiovascular disease in individuals with type 2 diabetes and hypertension in urban parts of San José, Costa Rica and Tuxtla Gutiérrez, Chiapas”. The intervention project, funded by the National Heart Lung and Blood Institute of the United States, aims to adapt and pilot test a cardiovascular risk reduction health education intervention model at primary health care centers. The two sites were selected because of already existing infrastructure and programs for patients with these chronic conditions. The purpose of the FGDs was to gain an understanding of patient knowledge and perceptions of disease in order to adapt the health education intervention that had previously been implemented elsewhere to each of the two populations.

The study had ethical approval from the institutional review boards of the RAND Corporation, the Institute of Nutrition of Central America and Panamá, the University of Arts and Sciences of Chiapas, the University of Costa Rica, and the CCSS.

### Population

The selection criteria for participating in the focus group discussions were: adults residing in each of the two cities with a confirmed diagnosis of arterial hypertension and/or type 2 diabetes; pregnant women were excluded from participating. Participants were recruited primarily by referral from health center providers at the public health centers and others with one or both of the conditions interested in participating in FGDs contacted the research team directly after seeing fliers posted in the waiting areas of the public facilities. In Costa Rica, participants were recruited through health centers of the Caja Costarricense de Seguridad Social and in Tuxtla Gutiérrez, Chiapas participants were referred by providers working at health centers of the Secretary of Health insured through a program called *Seguro Popular*, or popular insurance, that covers about a quarter of the population in Tuxtla. All potential participants were offered information about the research and those who were interested granted oral consent to participate.

### Data collection

A total of 12 focus group discussions were conducted: six in San José, Costa Rica and six in Tuxtla Gutiérrez, Chiapas, México. Six to eight people participated in each of the focus groups and a total of 12 focus groups were conducted. A script of questions was used for all FGDs and each one was conducted by members of the research team and was recorded using a digital recorder and a note taker documented observations. The FGDs explored participants’ perceptions of: the magnitude and symptoms of the conditions, their impact on families and communities, risk, prevention and treatment, existing infrastructure and services, and patients’ challenges and facilitating factors, and recommendations. All audio files were transcribed.

### Data analysis

We used a content analysis approach to identify themes related to factors that facilitate and prevent patients’ involvement in their disease management, focusing on a subset of relevant questions. All transcripts were entered in to Atlas.ti version 6 (2011) qualitative data management software (Scientific Software Development, Berlin, Germany) and were coded by two Spanish-speaking investigators using a pre-defined list of codes. The coded transcripts were then reviewed by two other members of the research team for consistency in coding and the team as a whole resolved differences through group discussion. The codebook was updated with new codes that emerged from the text during the process.

Then, after reviewing the text and grouping the codes into categories, we organized patient perceptions of disease into a table on patients’ acceptance of the disease informed by the stages of change of the Transtheoretical Model and contrasted factors that facilitate and prevent patients’ management of their disease. Quotes that represented each of the stages in participants’ phrasing were selected and are presented in the first column of the results table. How commonly the stages were referred to in the FGDs is presented. We also identified cross-cutting themes that were not linked to any one specific stage. As the intervention study for which focus group discussions were conducted in a formative phase, was a region-wide primary care intervention, we combined the analysis of the two sites, with an interest in finding differences and similarities in San José and Tuxtla Gutiérrez. We were particularly interested in commonalities in order to inform continued studies aimed at improving the provision of chronic care and cardiovascular risk reduction in the broad Mesoamerican region.

We then reviewed the transcripts again to confirm our findings and identified at least one quote that illustrated the themes in the data. Quotes were translated into English by bilingual authors and were edited only for ease of reading.

## Results

Twelve focus group discussions were conducted with a total of 70 adults with type 2 diabetes and/or hypertension. Thirty-eight adults diagnosed with type 2 diabetes or arterial hypertension, or both conditions, between the ages of 30 and 70 years of age participated in six FGDs in San José, Costa Rica. Thirty-two patients with one or both conditions between the ages of 37 and 73 years participated in six FGDs in Chiapas.

Without intentionally asking participants to describe the current stage in which they found themselves in managing their diabetes and/or hypertension, the different stages emerged in the discussions. In the following table we present representative quotes that capture the stages of change of FGD participants. And for each stage, we summarize the primary barriers and facilitating factors that were mentioned, recognizing that they vary depending on the extent to which a person living with hypertension or diabetes has taken an active role to promote change (Table 1).

**Table 1:** Reported barriers and facilitators of patients’ self-management organized in the stages of change framework

**Stage of change**	**Barriers to patients’ self-management**	**Factors that facilitate patients’ self-management**
**Pre-contemplation**	• Does not accept the diagnosis or hopes it will go away.	• Inclusive, horizontal communication.
*I haven’t suffered from it, but it appears that I am going to suffer from high blood pressure, when they take me in to check my blood pressure, yes it is high… (Chiapas, Female)*	• Lack of information about the disease.	• The primary health care institution is accessible (frequency of appointments, timing, publicly-funded).
*“I battled a long time with my husband about hypertension, and so we argued about him taking his pills, and he said “-ay! What for? if we all have to die from something…”* (San José, CR, Female)	• Does not feel any symptoms.	
	• Vertical communication between the provider and the patient.	
	• Poor eating habits and limited funds for healthy food.	
**Contemplation**	• Previous negative family health care experiences with hypertension or diabetes.	• Previous positive relative’s care experience with hypertension or diabetes.
*The doctor tells us that we should take care of ourselves, what we should do, she gives us appointments and she helps us. (Chiapas, Mexico Female)*	• Difficulties negotiating between work, family, and health care commitments.	• Guidance from the primary care provider that allows patients to express how they feel about their disease.
**Preparation**	• Negative perception of healthy food as being expensive or does not fill you up.	• Feeling of urgency to begin to take care of oneself.
*Having “sugar” is sad, I have high blood pressure, I have a warning, now it is up to me. (Chiapas, Mexico, Female)*	• Difficulty adhering to treatment (lack of medicines, lack of or infrequent follow-up).	• Community educational sessions.
	• Green spaces are not accessible.	• Family support.
**Action**	• Difficulty losing weight.	• Accessibility of the medical care system.
*Now I love myself, I am going to stick to my diet, and my medications, I have to lose 8 kilograms. (Chiapas, Mexico, Female)*	• Medication stock-outs.	• Humane, compassionate care is encouraging.
	• Difficulty controlling what food to eat/compulsive eating.	• Organized walking groups.
	• Additional health conditions.	
	• Not seeing progress/not having a record of change.	
	• Taking care of other family members.	
**Maintenance**	• Difficulty keeping track of multiple medications.	• High self esteem, self efficacy.
*Yes, every three months my doctor sees me in the clinic, weighs me, tells me you take this and that and gives me a prescription, it keeps me in control. (San José, Costa Rica, Male)*	• Health care appointment becomes routine without new information or educational processes.	• Strict health center attendance is a requirement for national insurance.
		• Staying calm and limiting stress.

The following two quotes refer to barriers that patients have related to lack of information that patients have and patient-provider communication. The first quote is a man in a stage of maintaining his disease but he has found it to be routine and is reflecting on stopping his medication to see how his body would react.

“The problem is that there is not information about whether it is developing positively or negatively, so one keeps taking pills and pills and pills but without really knowing if it is okay to stop, what do I know, one month to see what the response is. To see how your body responds, if problems return. I have been taking pills every day for four years for hypertension, diabetes, triglycerides, always the same medications, so then I don’t know if I should stop taking them or keep taking them because they always give me the same prescription.” (San José, CR, Male)

This woman in Chiapas prefers the experience of talking with fellow patients and information exchange to the unidirectional communication that she has experienced during clinic visits.

“However, there isn’t somebody there, like now in this case that we are talking and we are informing ourselves a little about the disease, I haven’t seen, that they approach us and they say come to a talk; but, the doctor does give you a talking-to: take care of yourself, get exercise.” (Chiapas, female)

A barrier to disease self-management that participants identified is having an additional health condition. A woman from Chiapas who is actively working on losing weight is frustrated because she does not see progress in weight loss despite improved habits because of taking treatment for another condition. Similarly, other participants refer to depression and physical limitations as barriers they face while actively trying to change behavior.

“…I am on a treatment, on a hormonal treatment because I have endometriosis and my doctor has told me that because of the medicine, I can’t lose weight, and now I don’t get hopeless anymore, but at the beginning I was on my diet eating grilled chicken, fish, fruits and vegetables and nothing, I didn’t lose any weight. So I said to the doctor ‘why can’t I lose weight and I even feel like I am gaining but it isn’t possible if I have been taking care of myself!’ And he says to me, ‘no, don’t lose hope what you need to do is maintain your weight and not gain more…’ sometimes I do lose hope and I say to myself if I am going to die I will die happy… (Chiapas, Female)”

“But then I, listening to what they are saying about depression, I sometimes feel alone, despite my sisters coming over, my son comes and they stay for a day with me, with the little one and his wife, and there are eight of us and we are very caring, they are all very good with me, but I feel alone because I miss my dad and so I, just hearing any little thing, it makes me want to cry and sometimes I spend as many as two days crying.” (San José, Costa Rica, Female)

Differences in disease type and time living with the disease can influence the extent to which patients are ready for change, how the disease manifests itself, and the interaction with their providers.

A woman from Costa Rica describes how she felt early on in pre-contemplation when she hoped that the disease would disappear but now has accepted it and manages it despite having moments when she feels down.

*“… they didn’t explain to me very well about this disease, but instead afterward I let it go, I thought it was like a fever that was going to go away (you hear somebody laughing) and then it wouldn’t bother me anymore… but now I know that I have to live with this disease, it is hard, because he, he is my husband but sometimes he says to me, ‘you are angry about everything’. No, it is just sometimes you get like you don’t want to talk about anything, you don’t want to know anything about the world, um, that is what happens to me, I don’t know about other people with this disease, um and I say to him, please understand me…(San José, Costa Rica, Female)*”

Below is a quote from a woman in Costa Rica who has lived with the disease for a long time and is trying to maintain her routine but at the clinic she feels that she is met with a provider who expects no changes in her condition and prescription.

*“…it is kind of like a routine, you know why? I have had high blood pressure for a long time, since I was 29 years old, and I should say that since I was about 32 years old I have been taking the same medications. Um … it is like drinking water. And I simply go and see, what did they give me? Um, so, oh God, one time I went in to a clinic, here in this health center, when I went in to the clinic there was a doctor and she said to me: ‘Yes?, I already have everything ready, here is your prescription’. She had everything ready, the prescription, and I froze! …, and I say but what is this, my God, like a bank! Ah, here take your change, … she had everything ready for me, … and so I said: what can I say?* (*San José, Costa Rica, Female*).”

### Factors that transcend individual patient self-management

In addition to finding quotes or codes that were more closely linked to one of the stages in the trans-theoretical model, we also encountered that there were a number of themes that were repeated that presented issues across stages, and that transcended individual patient self-management. The factors that we identified are: financial difficulty, gender, faith, and family.

### Financial difficulty

Financial difficulty was mentioned as a barrier to disease management - primarily in Chiapas - and was discussed as presenting an impact for the whole family. Financial concerns were expressed in terms of the cost of not being able to work because of illness, the expense of medications and exams, the higher cost of healthy foods, and the cost of caring for the person who is sick.

Even though participants in this study were recruited at health centers in which they receive care through public insurance, they discussed having to pay for extra services. And in Chiapas participants mentioned that it is often a problem that the health center they go to does not have medications available and they have to purchase them.

“Food is expensive; sometimes it isn’t possible to follow a diet, money makes the difference. I am in charge of my household, I am both mom and dad.” (Chiapas, Female)

“…well everything depends on the person, yes, if the person who works can’t bring money home well … It affects everyone not just the person with the disease.” (Chiapas, Female)

“I was used to not having breakfast because I started work at 6 am and with the rush I did not eat breakfast, and then I didn’t go out to not spend, I was economizing. I would hold off until 11 when I would get lunch… and I started to have sugar lows… and I am a single mother and I economized to the maximum to give my kids what they need.” (San José, Costa Rica, Female)

“When they (the health center) have medicines they give them to us and when they don’t we have to buy them …you have to buy medicines and it is an expense that you don’t have in mind. (Chiapas, Male)”

### Faith

Another cross-cutting factor that was mentioned by focus group participants in Costa Rica is faith in God as the primary facilitator of disease management.

“I ask God every day to keep me alive, so I can know my great-great-grandchildren, because I already know my great-grandchildren.” (San José, Costa Rica, Female)

“Thanks be to God I don’t have high blood pressure or diabetes. Blessed be God! I only have weak knees. My knees were really bad but God cured me some time ago, and now I am walking… so now I have muscle pain from walking, because I was in a wheelchair for 6 years. Thanks be to God.” (San José, Female) (It should be noted that while the patient states that she does not have high blood pressure or diabetes, she does indeed have at least one of the diseases and is in denial.)

### Gender

Another cross-cutting factor that emerged from the focus group discussions is gender, and was mentioned more in Costa Rica. The female role is often linked with caring for other family members as is presented in these quotes and represents a challenge for patients taking care of themselves. Being a single mother was also mentioned by a number of participants as an added difficulty for women.

“I take care of my father, because I am the only one of the sisters at home with him, and single. And for a while my dad was sick, with a bad heart and diabetes […], and I stopped taking care of myself, I stopped walking, it made me really anxious to see him sick, every day. […] so, for a long time I took care of him and I stopped taking care of myself, and I started getting fat again after losing a whole lot of kilograms” (San José, CR, Female)

“You take care of your husband but you don’t take care of yourself!…” (Chiapas, Female, paraphrasing her doctor)

“… and men are so negative about going to the doctor, right, a few of them are accessible but most are not, most of them don’t want to go … and it was a question of me having to tell him every day: take them, take them! I want to see you taking your pills.” (San José, CR, Female)

### Family support and how the family is affected

Finally, family support is an important factor in being able to stick to a regimen or not.

“Help from the family is very important, that children and the family understand that one cannot, or at least now my daughters avoid a lot of foods. I have trouble with desserts, all cakes and baked goods and bread and I used to make them at home, I used to make a lot of cakes at home. So now they understand that we cannot have desserts at home, they avoid buying them and bringing them home. Sometime they do, but they bring me a tiny piece because they tell me mamita because I know that you like it a lot, but I am only giving you a taste, just a little taste not to fill you up, and when they get paid their salary they go and buy splenda and one of those jams for diabetics and they buy whole wheat bread (San José, Costa Rica, Female).”

As presented in the focus group discussions, often both in the couple have a chronic illness and they become mutual caretakers:

“*Look, when you were sick – I tell him – you had somebody to help you, now it is both of us by ourselves, we do not have anybody else to help us, so we have to help one another*…(*San José, Costa Rica, Female*)”

Some participants did describe that they did not receive support from family members in actively managing their disease and sometimes found that they did better by not leaning on their families.

“*You have to learn to be healthfully selfish and put limits or what should I say… with food I do not have problems because I cook without salt and if they want to add salt to their food then they can add it but they didn’t understand and … but yes it (the family) helps a lot it has to help a lot because what if it is only causing you problems? To my two eldest kids, I stopped talking to them I don’t remember how many months so they wouldn’t say anything to me to avoid stress. Now they understand it…*”

## Discussion

Our study found that patients with diabetes and hypertension are extremely varied, with individuals falling at different stages on a spectrum of their readiness to change, and with barriers and facilitating factors varying based on their acceptance of the disease and their intent to change. Some participants in this study directly deny having the disease, although it was one of the inclusion criteria for the study, while others consider that their disease is in a manageable state.

A number of the facilitating factors and barriers to patient’s disease self-management were related to interfacing with the health center. Providers have the opportunity to orient their advice and the way in which they impart information to patients based on the stage that each patient is in. Even those who had the disease for many years considered their time with their provider to be important, although in some cases the clinical encounter had turned in to just a routine.

Disease self-management, and the stage at which each individual is situated should be understood in a broader family and societal context. The role of the family, financial considerations, gender differences and faith were all identified at this broader level. For example, as identified in this study and found elsewhere with respect to gender differences conditioned by society, women often take on the role of caregivers for others as is societally accepted [27] whereas men view barriers to disease self-management primarily in terms of their work responsibilities [28].

While health care providers are not positioned to intervene at this broader level, they can inquire about these factors to understand the resources patients have available to them, and recognize that there may be additional complications to consider. Specifically, in the case of family members, family physicians may be well-positioned to promote a kind of family-supported change and self-efficacy, especially in settings where families play a larger role.

While not the focus of this study, one element that may be important is the different emphasis that participants place on some barriers and facilitating factors in the two sites, because of differences in the health care system to which they have access. In comparing perceptions in the two settings, financial difficulty was expressed more in Chiapas as a problem than in Costa Rica, which may be due to the overall economic situation or may have to do with out-of-pocket costs that they have to cover. That said, in the Mesoamerican region, these two settings offer more services to patients with hypertension and type 2 diabetes than what is available in other lower-resourced countries. In Chiapas, check-ups and medications are offered free-of-charge but fewer tests and medications are covered as compared to what is included in the basic package offered through the Costa Rican social security health care system.

This study has a number of limitations. The FGDs that serve as the data source for this analysis were conducted with the primary objective of orienting and adapting a primary care cardiovascular disease risk reduction intervention to San José, Costa Rica and Tuxtla Gutiérrez, Chiapas, México. As such, it was not possible for our research team to follow-up with additional interviews or questions to go in to more depth on topics that emerged during the analysis. Another limitation is that people with disabilities related to their disease - such as an amputation or loss of sight -- did not participate in the FGDs; had they participated, they would have likely presented other perceptions of barriers and facilitating factors to disease management.

Participants were included in the FGDs regardless of disease type, gender, time that the person had been living with the disease. A future analysis that could provide valuable information is an analysis of the difference in disease management between patients with arterial hypertension and diabetes. Also in the future it would be interesting to separate men and women. Although a benefit to having them together in the same focus group was that both groups were able to hear some of the topics that might not have otherwise been raised such as suspecting infidelity and differences in service use and disease self-management between men and women.

## Conclusion

In sum, this study presents an analysis of patient perceptions of barriers and facilitating factors to disease self-management in urban San José, Costa Rica and Chiapas, Mexico, two settings for which there is limited published information available.

Patients with diabetes and hypertension in this study present a range of readiness to take on disease self-management, and barriers and facilitating factors that they discuss differ based on the stage in which they are currently situated. All participants in this study were diagnosed with either hypertension or diabetes but some state that they simply do not have the disease, whereas others have fully recognized their disease and are actively working to promote changes in their lifestyle to manage it.

There is an opportunity to improve disease self-management at the primary health care level in these two settings by better understanding specific barriers and facilitating factors that individual patients face, based on their stage of change, and also through the recognition that there are factors that transcend individual-level self-management.

## Abbreviations

CCSS: Costa Rican Social Security Institute; EBAIS: Basic health attention teams; FGDs: Focus group discussions.

## Competing interests

The authors declare that they have no competing interests.

## Authors’ contributions

MPF conceptualized the article, analyzed data, drafted the manuscript, and coordinated review of co-authors. NAM conducted focus group discussions, transcribed and analyzed data, contributed to writing, and reviewed and revised the manuscript for intellectual content and accuracy, and gave approval of the final version for publication. LP transcribed and analyzed data, contributed to writing, and reviewed and revised the manuscript for intellectual content and accuracy, and gave approval of the final version for publication. CMM contributed to writing and analysis, reviewed and revised the manuscript for intellectual content and accuracy, and gave approval of the final version for publication. SM participated in analysis, reviewed and revised the manuscript for intellectual content and accuracy, and gave approval of the final version for publication. HM contributed intellectually to the article, participated in analysis, reviewed and revised the manuscript for intellectual content and accuracy, and gave approval of the final version for publication. All authors read and approved the final manuscript.

## Pre-publication history

The pre-publication history for this paper can be accessed here:

http://www.biomedcentral.com/1471-2296/14/131/prepub
